# Influence of early stress on memory reconsolidation: Implications for post-traumatic stress disorder treatment

**DOI:** 10.1371/journal.pone.0191563

**Published:** 2018-01-19

**Authors:** Hélène Villain, Aïcha Benkahoul, Philippe Birmes, Barbara Ferry, Pascal Roullet

**Affiliations:** 1 Centre de Recherches sur la Cognition Animale, Centre de Biologie Intégrative, Université de Toulouse, CNRS, UPS, Toulouse, France; 2 Toulouse NeuroImaging Center, Université de Toulouse, Inserm, UPS,Toulouse, France; 3 Centre of Research in Neuroscience Lyon—UMR CNRS 5292—INSERM U 1028—Université Claude Bernard Lyon 1,Lyon, France; Harvard Medical School, UNITED STATES

## Abstract

Post-traumatic stress disorder (PTSD) is a common consequence of exposure to a life-threatening event. Currently, pharmacological treatments are limited by high rates of relapse, and novel treatment approaches are needed. We have recently demonstrated that propranolol, a β-adrenergic antagonist, inhibited aversive memory reconsolidation in animals. Following this, in an open-label study 70% of patients with PTSD treated with propranolol during reactivation of traumatic memory exhibited full remission. However, the reason why 30% of these patients did not respond positively to propranolol treatment is still unclear. One of the major candidates as factor of treatment resistance is the patient's early-life traumatic history. To test the role of this factor, mice with pre- or postnatal stress are being tested in fear conditioning and in a new behavioral task, the "city-like", specifically designed as a mouse model of PTSD. After reactivation of the traumatic event, mice received propranolol injection to block the noradrenergic system during memory reconsolidation. Results show that, in the “city-like” test, control mice strongly avoided the shock compartment but also the compartments containing cues associated with the electric shocks. Injection of propranolol after reactivation greatly reduced the memory of the traumatic event, but this effect was not present when mice had received pre- or postnatal stress. Moreover, propranolol produced only a very weak effect in the fear conditioning test, and never changed the corticosterone level whatever the behavioral experiment. Taken together our results suggest that our new behavioural paradigm is well adapted to PTSD study in mice, and that early stress exposure may have an impact on propranolol PTSD treatment outcome. These data are critical to understanding the effect of propranolol treatment, in order to improve the therapeutic protocol currently used in humans.

## Introduction

When a subject is exposed to a life-threatening traumatic event such as a terrorist attack, road traffic accident, personal assault, sexual abuse, natural disaster, or military combat, fear is the normal adaptive response [1]. In people who experienced such a traumatic event, the probability that posttraumatic stress disorder (PTSD) will develop varies according to gender and type of trauma; for example, the respective probabilities for men and women are 65% and 46% after rape, 2% and 22% after physical assault, and 6% and 9% after an accident [1]. Intrusion symptoms (e.g., recurrent distressing memories), avoidance of stimuli (e.g., efforts to avoid external reminders), negative alterations in cognition and mood, and alterations in arousal and reactivity (i.e., exaggerated startle response) usually begin within the first 3 months after the trauma [2]. Symptom duration varies, with complete recovery within 3 months in approximately half of cases [2]. Generally, 33% remain symptomatic for 3 years or longer, with greater risk of secondary problems [3]. In the United States, the projected lifetime risk for PTSD at 75 years of age is 8.7%; estimates are lower in Europe [2]. Significant variation in PTSD prevalence was found according to type of trauma, with highest prevalence for rape (17.4%), kidnapping (11.3%), other sexual assaults (11.0%), being beaten by a spouse or romantic partner (9.4%) and witnessing atrocities (8.7%), and the lowest for natural disasters (0.2%) and being a civilian in a war zone (0.7%) or a region with endemic terrorism (1.4%)[4].

A recent meta-analysis by Cusack et al. [5] showed that various therapies, such as exposure therapy (including prolonged exposure), cognitive therapy (including cognitive processing therapy, CPT), cognitive behavioral therapy (CBT), mixed therapies, eye movement desensitization and reprocessing (EMDR) and narrative exposure therapy (NET) significantly reduced PTSD symptoms (see [5] for a complete discussion of efficacy, comparative effectiveness, and adverse effects of these psychological treatments for adults with PTSD). Forty-three percent of patients do not respond adequately to second line treatments, using selective serotonin reuptake inhibitors (SSRIs) [6], and a recent meta-analysis concluded that SSRIs were only superior to placebo by a small effect size [7]. With only 50% of patients fully remitting [8,9] novel treatment approaches are needed.

Recent advances in neuroscience research have shown that, each time a memory is reactivated, the memory trace returns to an active labile state and must undergo reconsolidation in order to be maintained in the inactive stable state [10–13]. The majority of memory reconsolidation studies used protein synthesis inhibitors, and showed that, similarly to initial consolidation, memory reconsolidation requires new protein synthesis [12,13], notably for hippocampus-dependent memories [14–17].

Combining targeted memory retrieval with a reconsolidation impairing agent may disrupt unwanted memories, and constitutes a potential treatment strategy for patients suffering from psychiatric disorders that involve dysphoric memory, including PTSD [18]. However, the most common drugs used to block memory reconsolidation, protein synthesis inhibitors, cannot be used in humans due to safety issues [19]. Many animal studies therefore focused on other amnesic agents, such as the β-noradrenergic receptor antagonist propranolol that generated considerable interest recently [20–22]. Propranolol is a β-blocker which easily crosses the blood-brain barrier [23] allowing action at central level. In an early rat study [24], a single cue-induced reactivation followed by systemic administration of propranolol impaired memory in a passive avoidance task. Since then, many studies showed an amnesic effect of propranolol. For example, administration after retrieval disrupted contextual and auditory fear memory [25–27]. Propranolol associated to reactivation also dampened memory in a radial maze test [24], passive avoidance [20], cocaine and morphine conditioned place preference [28,29], and reward-related memory [30]. However, these studies yielded contradictory results, since no amnesic effect was found after propranolol treatment in passive avoidance [27], contextual conditioning [31], or object recognition [32]. In addition, the effects of propranolol seem to be greater during reconsolidation than during consolidation [20,24,25].

Based on animal research results, in 2008 Brunet et al. [33] developed a proof-of-concept study using post-reactivation propranolol in PTSD. More recently, in three independent studies, six brief trauma reactivation sessions under the influence of propranolol brought about large improvement in PTSD symptoms [34]. Moreover, physiological response during script-driven traumatic imagery continued to be reduced up to 4 months after treatment, demonstrating the durability of the effect [35]. In these experiments, 70% of patients with chronic PTSD treated with propranolol during reactivation of traumatic memory exhibited full remission. Despite these promising results, the reason why the other 30% did not respond positively to propranolol treatment remains unclear.

Some factors appear to be very important in the development of PTSD and may be factors of resistance to propranolol treatment efficacy in PTSD. Gender, type of trauma (multiple or personal vs. impersonal trauma), time between trauma and treatment, injury during trauma and early stress exposure may have an impact on PTSD treatment outcome [36], although results have sometimes been contradictory [37].

A growing body of evidence indicates a close relationship between the individual's trauma history and the development of PTSD. In animals, one of the consequences of early stress is a modification of neuro-endocrinological parameters, including lower baseline cortisol levels [38] with higher levels at the end of the stress response [39]. In PTSD patients, early stress is considered to be a major risk factor [40–42], and circulating levels of hormones involved in the flight-or-fight response to stress are altered, including lower baseline cortisol levels [43]. In general, women appear to have a more sensitized HPA axis with lower overall plasma cortisol than men [44]. A meta-analysis indicated that females with PTSD showed lower levels of basal cortisol than female controls [45]; no such difference was apparent between males. This may explain why women are more vulnerable than men to the development of post-trauma symptoms and take longer to recover from them [46]. For these reasons, only female mice offspring were used in this study, as only females display reduced cortisolemia subsequent to onset of PTSD [45,47].

These cortisol changes may reflect a pre-existing vulnerability trait that subsequently increases the probability of developing PTSD [48]. Pre-existing low cortisol levels were also observed in association with PTSD symptoms in motor vehicle accident victims [49], in response to natural disaster [47] and in rape victims [50]. Low cortisol levels were also found to be associated with parental PTSD in a study of descendants of Holocaust victims [51], and more recently in a cohort of infants of mothers exposed to the World Trade Center attacks during pregnancy [52].

General stress influences a wide range of learning and memory processes. Animal and human behavioral studies indicate that stress/anxiety can influence memory [53]. Although the precise mechanisms underlying the effects of emotional arousal on memory are not fully characterized, extensive evidence indicates that stress hormones, including catecholamines (e.g., noradrenaline) and glucocorticoids (corticosterone in rodents; cortisol in humans) play a critical memory-modulating role [32,54–58]. Interestingly, some data have suggested that the adrenergic and glucocorticoid systems interact to influence learning and memory processes. More precisely, some data obtained in pharmacology suggest that the effects of glucocorticoid system activation or blockade on memory depend on concurrent noradrenergic activity [59–63].

Early stress generally results also in significant impairment of mnemonic function in adulthood [64–67], and the number and the chronicity of the early stress episodes greatly influence the degree of memory deficit [67,68]. Animal studies confirmed the deleterious consequences of early stress on general behavior and on aversive or spatial memory in adulthood [69,70]. Prenatal stress induced an increase in anxiety and depressive behavior [71,72] and a clear decrease in mnemonic function, as observed in contextual and spatial memory [73–75]. These memory deficits were also observed in animals that received aversive electric foot-shocks in the second and third week after birth [76,77] or when pups were separated early from their mother [78].

As early life stress affects long-term memory and constitutes a potential risk factor for developing PTSD, it may also be a factor of resistance to propranolol treatment in PTSD. Childhood victimization is indeed one of the negative prognostic factors for psychiatric treatments, and specifically in adults with PTSD, where history of significant childhood sexual abuse is a negative predictor of pharmacotherapy outcome [79]. Moreover, repeated severe trauma during development may induce a more intricate clinical profile, called complex PTSD, which may contribute to poorer treatment response [80].

In order to determine the effects of early stress on the development and treatment of PTSD, the present study investigated the influence of pre- and postnatal early stress on the traumatic PTSD-like memory and on the efficacy of propranolol in reconsolidation therapy. Although common behavioral tests used in animals provided interesting insights into PTSD processes, they are not specifically designed to model the pathology. For example, in mice, the "fear conditioning" behavioral test, which associates an aversive event to a very simple context (generally a small 20×20 cm box with colored stripes walls), is very far from real complex contexts in which traumatic events occur in humans, such as a concert hall or city center, where they are hundreds of different cues (visual, auditory, olfactory, etc.). Moreover, flashbacks related to this event usually arise not when patients are in exactly the original context, but rather when a feature of the environment reminds them of the traumatic event (i.e., windy days after a hurricane)[2]. For these reasons, we felt it was important to develop a new behavioral test designed to mimic the situations encountered by patients as closely as possible.

To this end, a new “city-like” behavioral device in which mice could move around in different types of contexts was used to induce conditioned fear of one particular context. Then, fear-conditioned memory was partially reactivated by presenting some of the cues that were present in the conditioning context during acquisition, but in a new environment. Animals were injected with propranolol just after partial reactivation of the traumatic event, in order to test the role of the noradrenergic system during memory reconsolidation. Because prenatal stress is a major PTSD risk factor [40–42] and postnatal stress (such as sexual abuse in childhood) has been shown to be a negative predictor of pharmacotherapy outcome in PTSD [79], pre- and postnatally stressed mice were used in this new task. The effects of pre- and postnatal stress on propranolol efficacy to block memory reconsolidation were also evaluated in the classic fear-conditioned paradigm which is commonly used as a PTSD model in animals, in order to highlight the positive potentialities of our new task.

These results are of importance because, if perinatal stress decreases or reverses the propranolol effect in adults, the appropriateness of the new propranolol therapy for PTSD patients that suffered perinatal stress could be put into question. At all events, the project has the overall aim of improving understanding of the effect of propranolol in traumatic memory reconsolidation, and will provide new insights into the development of innovative approaches to PTSD treatment.

## Materials and methods

### Subjects and settings

A total of 158 adults (8 week-old) and 32 young (4 week-old) females CD1 mice born in the animal facility were issued from matings between CD1 mice (IFFA Credo, Lyon, France). Animals were housed in groups of four to six in standard breeding cages placed in a rearing room at a constant temperature of 25 ± 1°C under diurnal condition s (light–dark: 08.00 A.M.–08.00 P.M.), with food and water ad libitum. Behavioral tests were performed during the light period (between 8:00 A.M. and 3:00 P.M.). All procedures involving animals and their care are conformed to the institutional guidelines, which comply with international laws and policies (directive 2010/63/European Community) and have been approved by the ethics committee of the Paul Sabatier-Toulouse 3 University (C2EA-01 FRBT). Permission reference was EU0113 #2016041116577561. All other co-authors were under the responsibility of the former.

Briefly, after mating and parturition, dams and litters were left undisturbed until on postnatal weaning day (PND) 21. Then, all the young mice were segregated by sex. Males were culled and females from different lineages were mixed and housed in groups of four to six animals. The body weight of pregnant females and litters was measured every day at 9:00 A.M. to monitor pregnancy and litters growth. After weaning, the two groups of pups born from stressed and non-stressed mothers were separately housed in similar breeding cages.

### Overall experimental procedure

**Fig 1:**
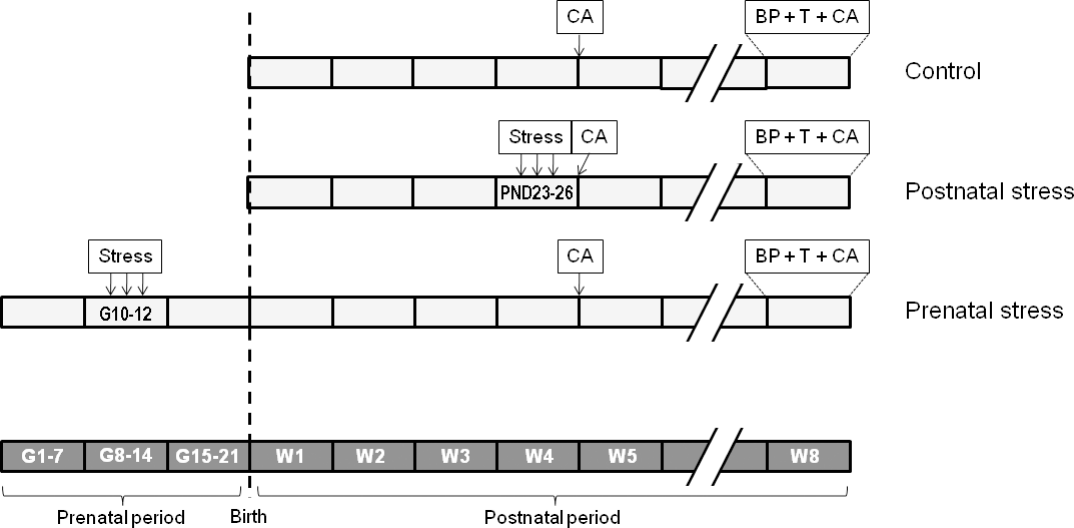
Overall experimental design. Stress: stress exposure; CA: corticosterone assay; BP: behavioral procedures; T: treatment; G: gestational day; PND: postnatal day; W: week.

The general procedure used in the experiments is shown in Fig 1. To study prenatal and postnatal stress influence on traumatic memory reconsolidation and on propranolol efficiency to modify this traumatic memory, animals born from non-stressed mothers were divided in two subgroups: the first group (postnatal stress group, n = 54) was submitted to stress exposure procedure (cf. the “postnatal and prenatal stress exposure procedure” section) after weaning from PND 23 to PND 26; the second group corresponded to the no-stress Control group (n = 48); the third group (prenatal stress, n = 56) corresponded to offspring of dams stressed from G 10 (gestational day) to G 12. Mice in this group did not receive any additional stress after birth. Careful attention was paid to the constitution of all experimental groups, and different mothers (at least 4) were used to obtain offspring in each group. Finally, we verified that the results in each group were not due to any effect of “mother” as factor.

All groups were tested in the “city-like”, the contextual and cued FC paradigms 8 weeks after birth. Thirty min after the end of the behavioral tests, animals in the different experimental groups were sacrificed and blood was collected to measure plasmatic corticosterone levels. Basal serum corticosterone concentration was measured in 4 week-old groups of mice in the three experimental conditions: no-stress (n = 12); postnatal stress (n = 9) and prenatal stress (n = 10).

### Postnatal and prenatal stress exposure procedure

The procedure consisted in exposing the mice to three types of stress procedures each day for three days, at the same time schedule but in a random order. The first procedure corresponded to a restraint stress induced by keeping mice in a small cylindrical tube (3 cm in diameter and 11.5 cm long) for 2 hours. The second procedure corresponded to a forced swim stress induced by placing the mice in an opaque pool (20 cm in diameter and 21 cm long) filled with 20°C water for 20 min, at the end of which they were removed from water and dried. The third procedure corresponded to an electric foot-shock-induced stress. For this, mice placed on a grid floor made of 2 mm metal bars spaced 5 mm apart, in an unfamiliar box (13 × 8× 7.5 cm) and five brief foot-shocks (545 μA, 2 sec) separated by a 10 sec interval were delivered. Mice were placed in their respective home-cage for one minute between each procedure.

### Behavioral testing

#### City-like

**Fig 2:**
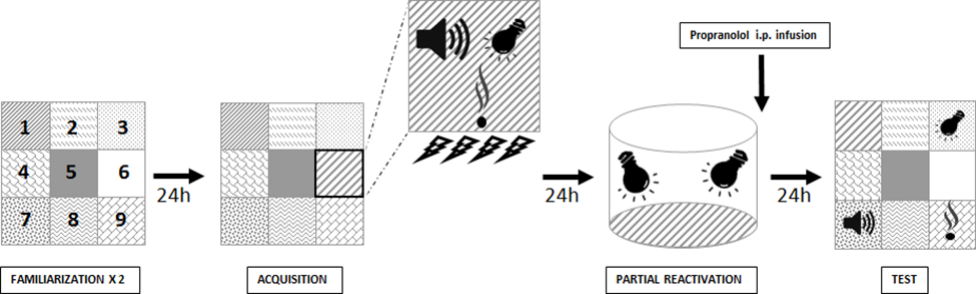
« City-like » behavioral task. Top view of the new behavioral device developed for the study of PTSD. The apparatus (1m × 1m) is divided in 9 distinct compartments that differ in the type of feature presented in each of them (i.e. self-sticking papers with different patterns). In this box, mice pass through the central compartment (position 5) to reach the others. Behavioral procedure consists in the succession of 4 experimental phases. Mice were first acclimated to the device for two days. Then, traumatic Acquisition took place on Day 3 and was partially Reactivated on Day 4. Traumatic memory was finally Tested on Day 5 by placing the mice in the “city-like” box where the shock-associated cues were presented individually in 4 distinct compartments. The testing box was finally constituted by 9 distinctive compartments: two compartments presenting the reactivated tactile (copper plate floor, position 1) and visual (light, position 3) cues; one compartment where mice received the shocks (position 6); three neutral compartments (position 2, 4, and 8) and two compartments presenting the two non-reactivated auditory (positions 7) and olfactory (position 9) cues.

To establish an animal model of PTSD, we developed a new behavioral paradigm called “city-like” consisting in the succession of four experimental phases in a box constituted by 9 different compartments (Fig 2). The Familiarization phase consisted of two sessions (Day 1 and Day 2) of 30 min during which the animals could explore the different compartments of the box. Each session was separated by an inter-trial interval of 24h. The Acquisition phase took place on Day 3, and consisted for the animal to associate the context of a particular compartment of the box with a negative event (unconditioned stimulus, US). For this, mice were placed in one compartment of the box (Shock compartment, position 6 on Fig 2) in which new visual (flashing white light), olfactory (a blotting paper soaked by 4 μL of octanol), auditory (a continuous high-pitched tone, 4100 Hz, 70 dB) and tactile (a copper plate) cues were added. Immediately after being placed in this compartment, mice received a series of four successive electric foot-shocks US (580 μA, 2 sec). On Day 4, a partial Reactivation of the context-US memory was achieved by placing the animals in a completely new context (opaque cylinder 20 cm in diameter and 20 cm long) in the presence of only 2 (visual + tactile) of the 4 cues previously associated with the US during the acquisition. During the Test phase on Day 5, mice were placed in the “city-like” box where each of the 4 cues previously associated with the US were presented individually in the 4 corner compartments of the device (positions 1, 3, 7 and 9 on Fig 2) where they stayed for a total of 30 min. To assess traumatic memory, the latencies to enter the Shock compartment (position 6 on Fig 2) and each corner compartments were measured and compared to the latencies to enter the 3 neutral compartments (position 2, 4 and 8 on Fig 2). Freezing time was measured in the Shock and Plate compartments (position 1, presenting the tactile cue) for which mice showed the highest latency before entering.

#### Contextual and cued fear conditioning

In order to evidence the evaluate the scientific benefit that we could gain from the new “city-like” paradigm compared with those classically used in the literature to study the processes underlying PTSD in animal models, three other groups of mice (no-stress, postnatal stress and prenatal stress) were tested in the fear conditioning (FC) paradigm. Briefly, mice were placed in a rectangular conditioning chamber (length 35 cm, width 20 cm, height 25 cm) with a stainless steel rod floor for 5 min and 30 sec. After a 2-min exploration period, a tone (85 dB) (conditioned stimulus, CS) was emitted for 30 sec and a foot-shock of 0.7 mA (US) was superposed to the tone during the last 2 sec. After 2 min, the same sequence was repeated.

In contrast with our city-like paradigm, reactivation of the traumatic memory took place twenty-four hours later in the acquisition context (conditioning chamber) where mice were placed for 15 sec without tone and shock [81]. The day after, contextual fear memory was measured by replacing both groups in this acquisition context during 4 min and the freezing time was measured. To test the fear memory to the tone CS (cue memory test), both groups of animals were placed in the new context 3 h later. Two min after, mice received a 2-min tone presentation and the freezing time was measured during the total duration of the test. Freezing behavior was defined as immobility except for respiratory movements.

### Drug and injections

DL-propranolol (10 mg/kg, Sigma, France), a β-adrenoceptor antagonist, was prepared in 0.9% saline and administered intraperitoneally at a volume of 10 mL/kg. Saline (NaCl group) or propranolol (Propranolol group) injections were performed immediately after the Reactivation phase of the two “city-like” and FC paradigms, which took place 24 h before the memory Test. Consequently, animals were never tested under propranolol influence but the day after injection, when the drug was no longer present in the organism.

### Determination of serum corticosterone levels

Corticosterone levels were measured in the three experimental groups 4 weeks after birth, and also 30 minutes after the end of the behavioral test during the 8th week. For basal corticosterone level determination, mice in each group were killed by cervical dislocation between 10:00 A.M. and 2:00 P.M. After decapitation, blood samples (1.5 ml) were collected in heparinized tubes. The samples were centrifuged at 3500 rpm for 15 min at room temperature to obtain serum that was quickly frozen and stored at -20°C until use. Plasma corticosterone levels were assayed using commercial radioimmunoassay kits (Corticosterone ELISA, IBL International GmbH; Corticosterone EIA Kit, Enzo Life Sciences) according to the manufacturer’s instructions.

### Data analysis

In this study, each animal was tested in only one behavioral paradigm and was submitted to one of the two treatments (propranolol or saline solution), thus all groups were independent. In the “city-like” paradigm, conditioned fear memory was estimated by measuring the latency of entering in each compartment during the test. The mean (± S.E.M.) latencies of entering the compartments (sec) was analyzed with a two-way analysis of variance (ANOVA), with Context (Shock, Plate, Odor, Light, Tone and Neutral compartments) as within subject factor and Treatment (Propranolol vs. NaCl) as between subject factors. Observation of the data showed that most of the mice in the Control group avoided to enter the Shock and Plate compartments and this resulted in a large number of ceiling latency values of 1800 sec (30 min corresponding to the total duration of the test). Therefore, the data were converted into ratios corresponding to the “(number of mice that avoided the Shock and Plate compartments during the 1800 sec total test period / total number of animals of the group) ×100”. The ratios calculated for each compartment were analyzed with a one-way ANOVA.

In the contextual FC task, the percentage of freezing, calculated as “(time of freezing / total time of the session) ×100” was analyzed with a one-way ANOVA. Serum corticosterone concentration levels (ng/ml) were expressed as mean + S.E.M. and analyzed with a one-way ANOVA. Post hoc Tukey comparisons were used to determine the source of detected significances in the ANOVAs when needed. A probability level of < .05 was accepted as statistically significant throughout.

## Results

### Propranolol effect in no-stress condition

#### City-like test

Analysis of the latencies to enter each compartment of the city like box during the Familiarization showed no statistical difference between NaCl and Propranolol groups. Also, the comparison of the latencies to enter the Shock compartment during the acquisition did not reveal any significant difference between the two experimental groups (see supporting information file S1 Dataset).

**Fig 3:**
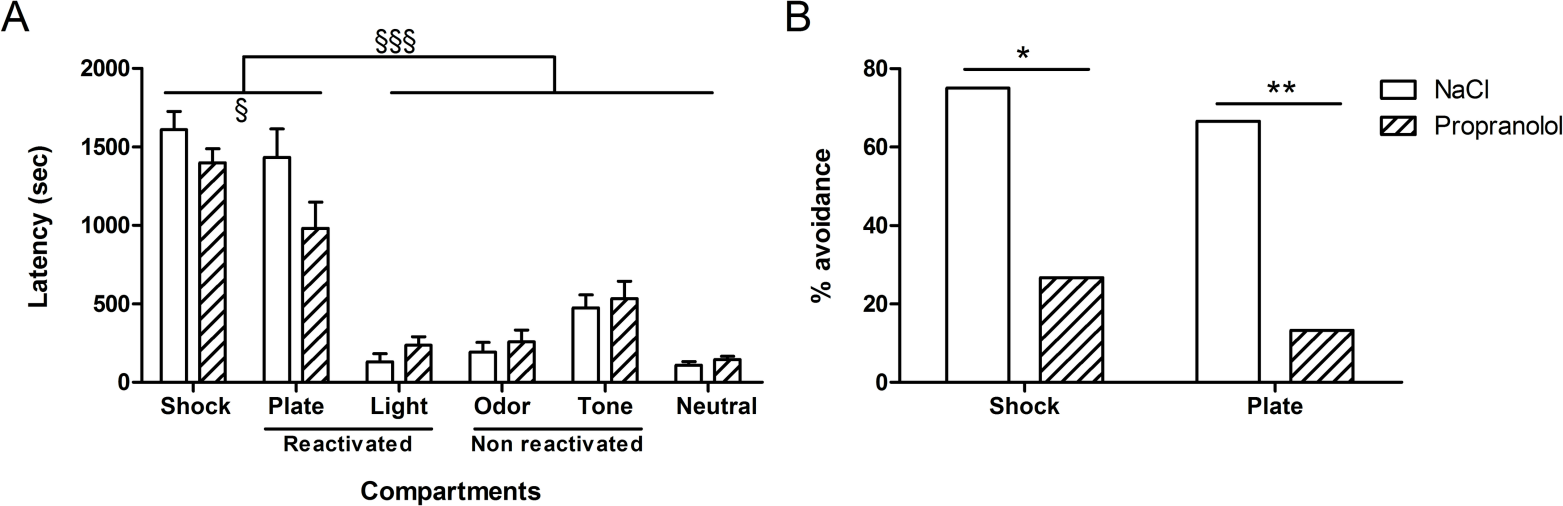
Effect of propranolol on reconsolidation of traumatic memory without any stress in the “city-like” paradigm. **(**A): Latencies (mean + SEM) to enter the different compartments of the “city-like” box. Shock; compartment where mice received the shocks during the acquisition (position 6 on Fig 2); R cues: compartments presenting the tactile (position 1) and visual (position 3) cues that were reactivated. NR cues; compartments presenting non reactivated auditory (positions 7) and olfactory (position 9) cues. Neutral: neutral compartments (position 2, 4, 5, and 8). (B): avoidance percentage corresponding to the proportion of animals that entered the Shock and the Plate compartments during the test. *: p < .05, **: p < .01 between NaCl and Propranolol groups in the “no stress” condition. ^§^: p < .001, ^§§§^: p < .001 between the different compartments. (NaCl n = 12; Propranolol n = 15).

The results obtained during the test in the “city-like” box for the various groups are shown in Fig 3. Fig 3A represents the mean latencies (± S.E.M.) to enter each compartment measured during the test session whereas Fig 3B represents the percentage of animals that avoided the Shock and the Plate compartments in each experimental group.

As shown in Fig 3A, the mean latencies to enter each compartment was similar between Propranolol and NaCl groups. However, the mean latencies obtained in each group differed depending on the type of compartment. Statistical analyses confirmed these observations and revealed no significant effect of Treatment on latencies to enter the different compartments [F(1, 150) = 1.30; p = .253], a significant effect of Compartment [F(5, 150) = 68.45; p < .001] and a significant interaction between both factors [F(5, 150) = 2.39; p = .043]. Post hoc comparisons showed only a marginal difference between Propranolol and NaCl groups in the latencies to enter the Plate compartment (p = .066). In addition, post-hoc tests showed that the latencies to enter the Shock and the Plate compartments were significantly higher than the latencies to enter the four other compartments (P < .001) in both NaCl and Propranolol groups. Post hoc tests showed moreover that the latencies to enter the Shock compartment were significantly higher than the latencies to enter the Plate compartment (p < .05).

Careful observation of the behavior revealed that mice avoided particularly the Shock compartment, where they received the shocks and the Plate compartment which contained the cue through which the shocks were delivered. The other compartments were never completely avoided and mice always ended up by entering them before the end of the session. Interestingly, while control mice (stress free) highly avoided the Shock and the Plate compartments, however, the few mice that finally (latency > 1500 sec, Fig 3A) entered these chambers during the test, went out quickly as showed by the time spent in each of them (Shock compartment: 13 sec; Plate compartment: 48 sec, see supporting information files).

As shown in Fig 3B, the percentage of mice that avoided the Shock and Plate compartments (referred to as “percentage avoidance”) obtained in the two experimental groups differed according to the type of treatment. Whereas almost all the animals in the control group avoided entering the two target compartments, the proportion of animals injected with propranolol that avoided these compartments was largely smaller. Statistical analyses confirmed these observations and revealed a significant difference in the percentage avoidance between the two compartments between NaCl and Propranolol groups (F(1, 25) = 7.51; p < .05 and F(1, 25) = 10.77; p < .01 for the Shock and Plate compartments successively). The differences between the latencies to enter the different compartment suggest that the associative strength between the tactile and the “location” cues with the shocks is higher than this of the auditory, visual and olfactory cues. Moreover, the comparison between the number of animals that avoided the Shock and the Plate compartments suggest that propranolol affected the processes underlying the reconsolidation of the traumatic memory.

#### Fear conditioning

**Fig 4:**
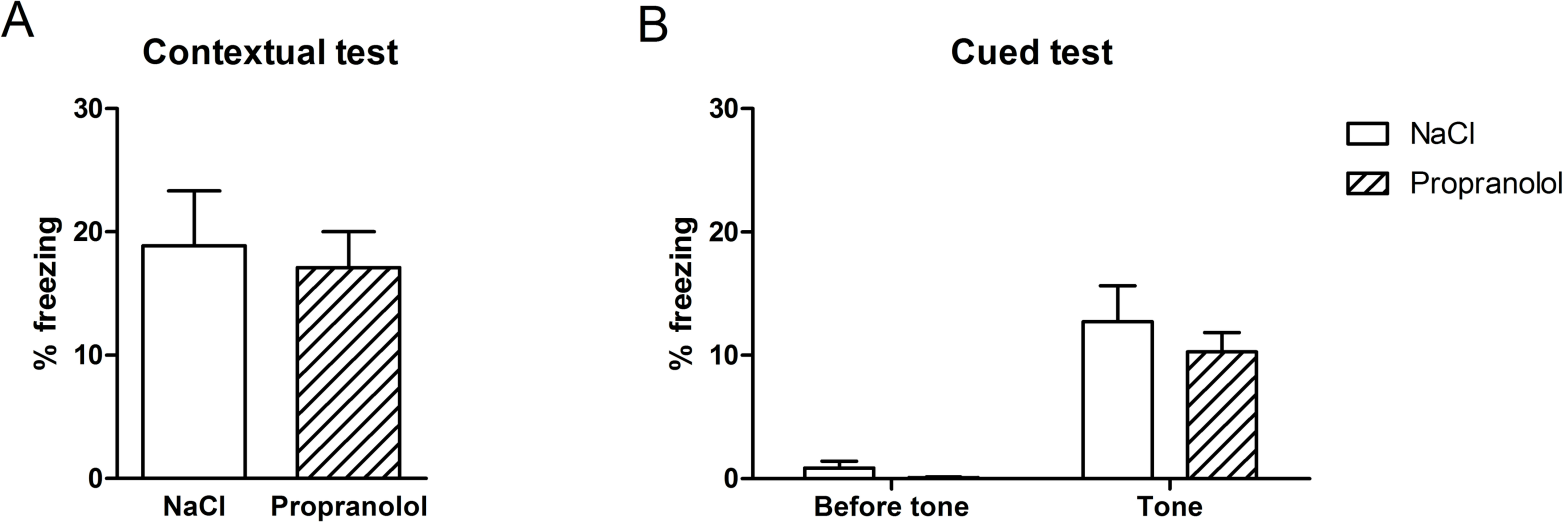
Effect of propranolol on reconsolidation of the aversive memory using the contextual and the cued fear conditioning in the no-stress group. Percentage freezing time (mean + SEM) during the contextual test (A) and the cued test (B). (NaCl n = 10; Propranolol n = 11).

The results obtained during the FC test in the various groups are shown in Fig 4. Fig 4A represents the mean percentage of freezing time (± S.E.M.) calculated in the two experimental groups during the contextual test whereas Fig 4B represents the mean percentage of freezing time (± S.E.M.) calculated before and after the presentation of the tone during the cued test for the two experimental groups.

As shown in Fig 4A, the mean amount of freezing measured in the acquisition context of FC was similar between groups suggesting that propranolol did not affect the reconsolidation of the context-US association during the reactivation phase of the FC. Statistical analyses confirmed these observations and revealed no significant effect of Treatment on the mean percentage of freezing time measured during the contextual test (F(1, 21) = .11; p = .742]. As shown in Fig 4B, the mean amount of freezing measured in the new context in the presence of the tone was similar between groups in both pre-tone and post-one conditions. While animals in both experimental groups showed no freezing before the presentation of the tone, the percentage of freezing increased following the tone presentation. Statistical analyses confirmed this description and showed that the percentage of freezing did not statistically differ between the Propranolol and the NaCl group in the two pre- and post-tone conditions (F(1, 21) = .85; p = 0.201 and F(1, 21) = .58; p = 0.454 respectively).

These results show that propranolol did not affect the processes underlying the reconsolidation of the conditioned auditory and contextual fear memories when injected immediately after the reactivation.

#### Conclusion for no-stress

These data suggest that, in the FC paradigm, β-adrenoceptor blockade did not influence the reconsolidation of the aversive auditory and contextual memories. The contradiction between these results and those obtained with the “city-like” paradigm suggests that classic FC paradigm does not allow to identify the involvement of the adrenoceptor system in the memory processes underlying the reconsolidation.

### Propranolol effect in postnatal stress condition

#### City-like

**Fig 5:**
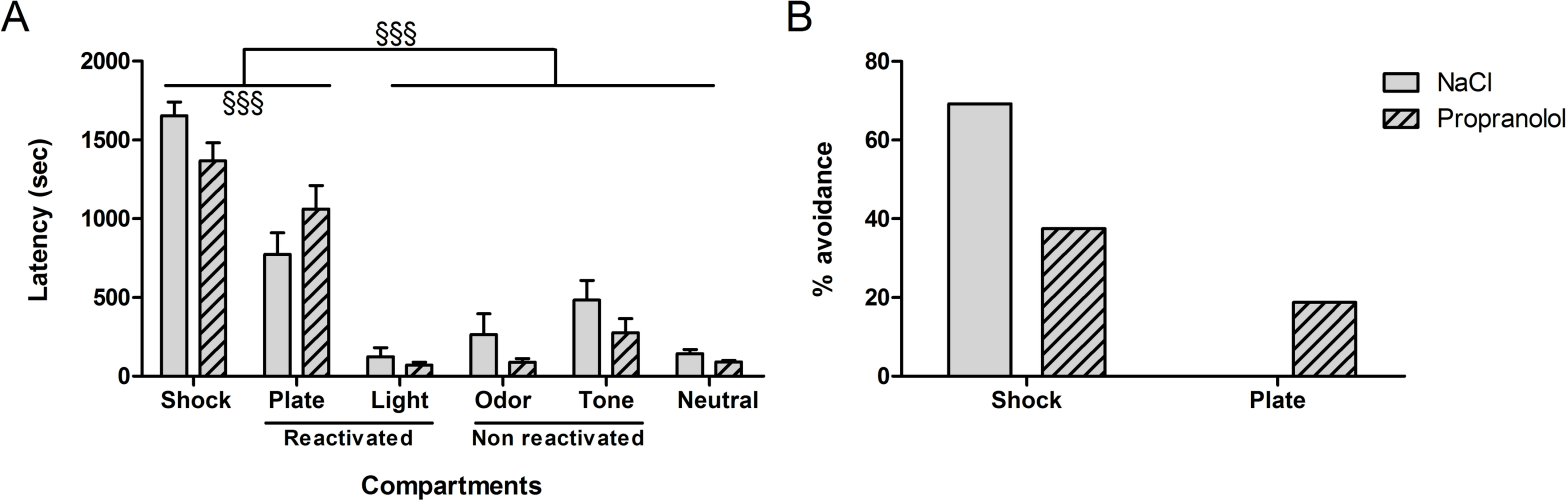
Effect of propranolol on reconsolidation of the traumatic memory using the “city-like” paradigm after postnatal stress. (A) Latencies (mean + SEM) to enter the different compartments of box. (B) Avoidance percentage corresponding to the proportion of animals that entered the Shock and the Plate compartments during the test. ^§§§^: p < .001 between the different compartments. (NaCl n = 13; Propranolol n = 16).

The results obtained during the test in the “city-like” box for the various groups are shown in Fig 5. As shown in Fig 5A, the mean latencies for entering each compartment was similar between Propranolol and NaCl groups. However, the mean latencies obtained in each group differed depending on the type of compartment. Statistical analyses confirmed these observations and revealed no significant effect of Treatment on latencies to enter the different compartments [F(1, 162) = 2.24; p = .136], a significant effect of Compartment [F(5, 162) = 73.13; p < .001] and a significant interaction between both factors [F(5, 162) = 2.31; p = .046]. Post hoc comparisons showed no difference between NaCl and Propranolol groups in the latencies to enter each compartment. In addition, post-hoc tests showed that the latencies to enter the Shock and Plate compartments were significantly higher than the latencies to enter the four others compartments (P < .001) in each group. Post-hoc tests showed moreover that the latencies to enter the Shock compartment were significantly higher than the latencies to enter the Plate (p < .001) compartment in both groups.

As shown in Fig 5B, the percentage of mice that avoided the Shock and Plate compartments (percentage avoidance) obtained in the two experimental groups differed according to the type of context. Whereas almost all the animals in the control group avoided entering the Shock compartment, they showed no aversion to the Plate compartment. However, statistical analyses failed to reveal any significant difference in the percentage of mice entering the two compartments between the NaCl and the Propranolol groups (F(1, 27) = 2.99; p = .095) and F(1, 27) = 2.79; p = .106 for the Shock and Plate compartments successively).

In conclusion, these results suggest that postnatal stress affected the avoidance level of the Plate compartment but did not influence the avoidance behavior of the Shock compartment. In contrast to Experiment 1, propranolol did not significantly affect this avoidance level but had only a modest impairment on the latency to enter the different compartments.

#### Fear conditioning

**Fig 6:**
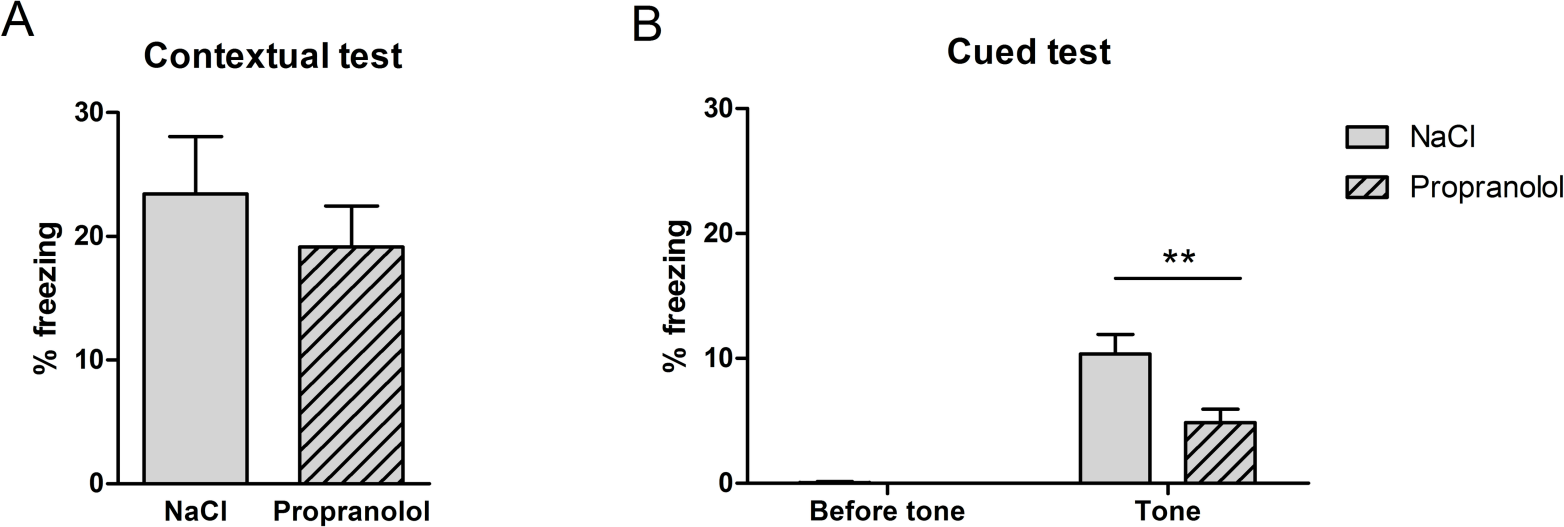
Effect of propranolol on reconsolidation of the aversive memory using the contextual and cued fear conditioning after postnatal stress. Percentage freezing time (mean + SEM) during the contextual test (A) and the cued test (B). **: p < .01 between NaCl and Propranolol. (NaCl n = 12; Propranolol n = 13).

The results obtained during the contextual and cued FC tests in the various groups of mice are represented in Fig 6. As shown in Fig 6A, the mean amount of freezing measured in the acquisition context of contextual FC was similar between groups suggesting that propranolol did not affect the reconsolidation of the context-US association during the reactivation phase of the contextual FC. Statistical analyses confirmed these observations and revealed no significant effect of Treatment on the mean percentage of freezing time measured during the contextual test (F(1, 23) = .58; p = .452). As shown in Fig 6B, while animals in both experimental groups showed no freezing before the presentation of the tone during the cued test, the percentage of freezing increased following the tone presentation and this increase was higher in the NaCl control group. Statistical analyses confirmed this description and showed that the percentage of freezing did not statistically differ between the Propranolol and the NaCl groups in the pre-tone condition (F(1, 23) = 1.08; p = .308) but differed between groups in the post-tone condition (F(1, 23) = 8.62; p = .007)

These results suggest that, in the FC paradigm, β-adrenoceptor blockade did not influence the reconsolidation of the aversive contextual memory and selectively affected the auditory conditioned fear memory. In contrast to the results obtained in Experiment 1, postnatal stress condition revealed the involvement of the β-adrenoceptor system in the reconsolidation processes.

#### Conclusion for postnatal stress

In the “city-like” test, propranolol induced only a small decrease in memory reconsolidation of the traumatic memory and had no effect on avoidance behavior. However, in the fear conditioning test, propranolol-treated mice displayed a significant decrease in freezing behavior in the cued test.

### Propranolol effect in prenatal stress condition

#### City-like

**Fig 7:**
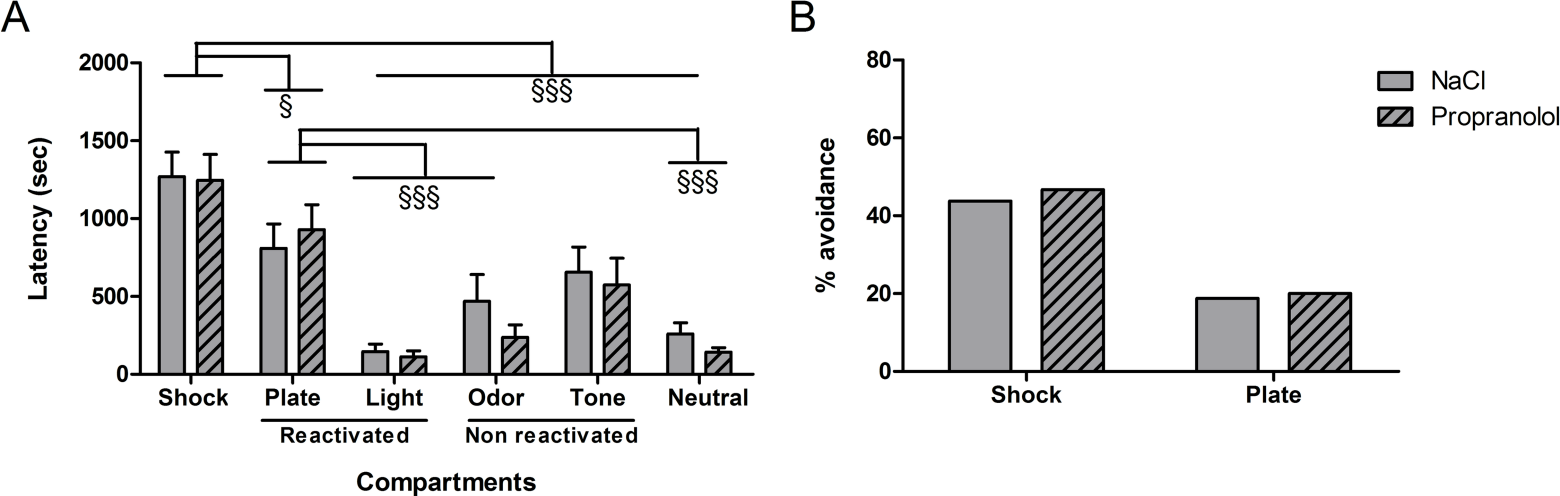
Effect of propranolol on reconsolidation of the traumatic memory using the “city-like” paradigm after prenatal stress. (A) Latencies (mean + SEM) to enter the different compartments of the box. (B) Avoidance percentage corresponding to the proportion of animals that entered the Shock and the Plate compartments during the test. ^§^: p < .001, ^§§§^: p < .001 between the different compartments. (NaCl n = 16; Propranolol n = 15).

The results obtained during the test in the “city-like” box for the various groups are shown in Fig 7. As shown in Fig 7A, the mean latencies to enter each compartment were similar between Propranolol and NaCl groups. However, the mean latencies obtained in each group differed depending on the type of compartment. Statistical analyses confirmed these observations and revealed no significant effect of Treatment on latencies to enter the different compartments (F(1, 172) = .65; p = .425), a significant effect of Compartment (F(5, 172) = 21.61; p < .001) and no significant interaction between both factors (F(5, 172) = .40; p = 0.855). Post hoc comparisons showed no difference between NaCl and Propranolol groups in the latencies to enter each compartment. In addition, post-hoc tests showed that the latencies to enter the Shock compartment were significantly higher than the latencies to enter the four others compartments (p < .001) and the latencies to enter the Plate compartment were significantly higher than the latencies to enter the Light, Odor and Neutral compartments (p < .001). Post-hoc tests showed moreover that the latencies to enter the Shock compartment were significantly higher than the latencies to enter the Plate compartment (p < .05). As shown in Fig 7B, the percentage of mice that avoided the Shock and Plate compartments obtained in the two experimental groups differed according to the type of context but not the type of treatment. Whereas half of the animals in the NaCl and Propranolol groups avoided entering the Shock compartment, the proportion of animals in both experimental groups that avoided the Plate compartment was smaller. However, statistical analyses failed to reveal any significant difference in the percentage of mice entering the two compartments between the NaCl and the Propranolol groups (F(1, 23) = .48; p = .492 and F(1, 23) = .15; p = .704 or the Shock and Plate compartments successively). In conclusion, these data suggest that prenatal stress affected the avoidance level for the Plate compartment. They also show that prenatal stress blocked the propranolol-induced impairment of memory reconsolidation of the trauma previously observed in the non-stressed mice.

#### Fear conditioning

**Fig 8:**
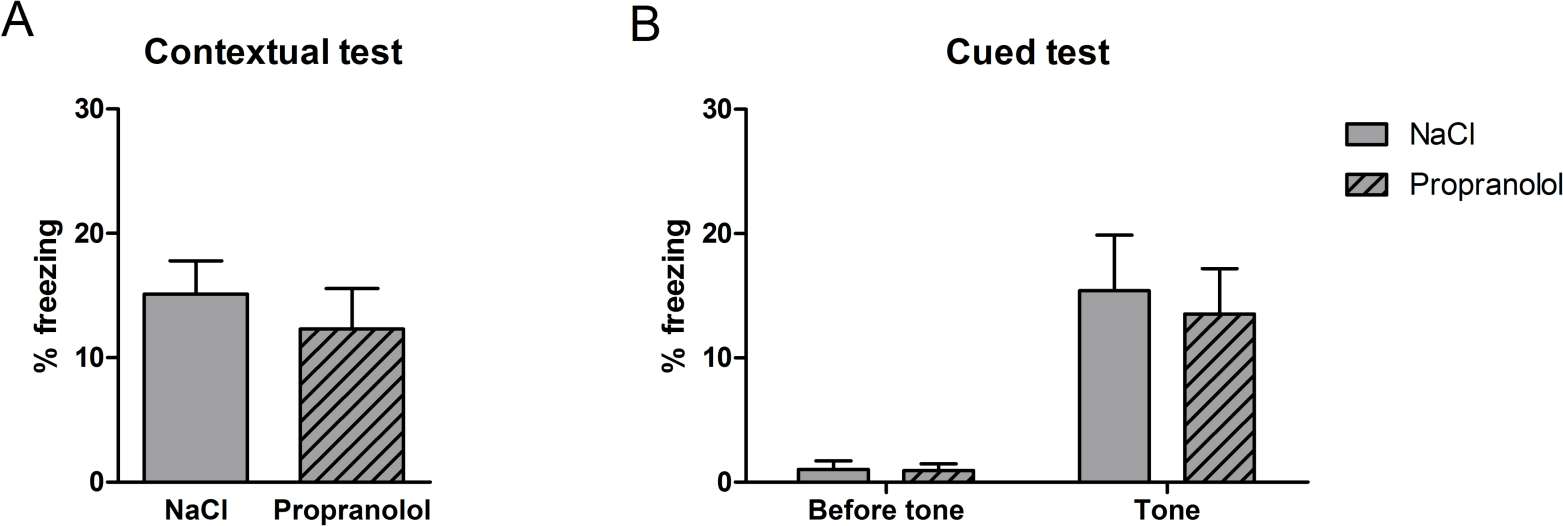
Effect of propranolol on reconsolidation of the aversive memory using the contextual and cued FC after prenatal stress. Percentage of freezing time (mean + SEM) during the contextual test (A) and the cued test (B). (NaCl n = 12; Propranolol n = 13).

The results obtained during the contextual and cued FC tests in the various groups of are shown in Fig 8. As shown in Fig 8A, the mean amount of freezing measured during the acquisition of contextual FC was similar between groups suggesting that propranolol did not affect the reconsolidation of the context-US association during the reactivation phase of the FC. Statistical analyses confirmed these observations and revealed no significant effect of Treatment on the mean percentage of freezing time measured during the contextual test (F(1, 23) = .13; p = .723). As shown in Fig 8B, while animals in both experimental groups showed no freezing before the presentation of the tone during the cued FC test, the percentage of freezing increased following the tone presentation. Statistical analyses confirmed this description and showed that the percentage of freezing did not statistically differ between the Propranolol and the NaCl group in the two pre- and post-tone conditions (F(1, 23) = .02; p = .885 and F(1, 23) = .11; p = .745 respectively).

These results show that prenatal stress did not affect the processes underlying the reconsolidation of the conditioned auditory and contextual fear memories. Moreover, the data show that propranolol did not affect the reconsolidation process when injected immediately after reactivation in prenatally stressed animals.

#### Conclusion for prenatal stress

Propranolol injected during reactivation did not affect aversive memory reconsolidation in prenatally stressed mice tested in the “city-like” test and the fear-conditioning test.

### General conclusion for the 3 stress conditions

The first finding demonstrated by the results is that propranolol-induced β-adrenergic system blockade immediately after reactivation in our “city-like” paradigm affected the reconsolidation of traumatic memory but not the reconsolidation of contextual FC. The second important finding is that prenatal and postnatal stress blocked the effect of β-adrenoceptor blockade on memory reconsolidation in the “city-like” paradigm; in contrast, propranolol injection affected fear-conditioned memory performance in the cued FC in post- but not in the prenatally stressed mice (see Table 1).

**Table 1:** Summary of the treatment effect (ANOVA) in the two behavioral tasks and in the three stress groups.

*TREATMENT EFFECT*	City-like			Fear conditioning	
	Latency	Avoidance		Contextual test	Cued test
**Control**	F(1, 150) = 1.30; p = .253	Shock	**F(1, 25) = 7.51; p < .05**	(F(1, 21) = .11; p = .742].	F(1, 21) = .58; p = 0.454
		Plate	**F(1, 25) = 10.77; p < .01**		
**Postnatal stress**	[F(1, 162) = 2.24; p = .136	Shock	F(1, 27) = 2.99; p = .095	F(1, 23) = .58; p = .452).	**F(1, 23) = 8.62; p = .007**
		Plate	F(1, 27) = 2.79; p = .106		
**Prenatal stress**	F(1, 172) = .65; p = .425	Shock	F(1, 23) = .48; p = .492	(F(1, 23) = .13; p = .723	F(1, 23) = .11; p = .745
		Plate	F(1, 23) = .15; p = .704		

Significant *P*-values are given in bold.

To shed more light on the effects of propranolol in the pre- and postnatally stressed animals, we next analyzed the different stress effects on the two main behavioral parameters impacted by propranolol injection i.e., percentage avoidance of the Shock and Plate compartment in the “city like” paradigm and percentage of freezing in the contextual and cue versions of the FC test.

### Effect of the stress conditions on the avoidance behavior in the “city-like”

**Fig 9:**
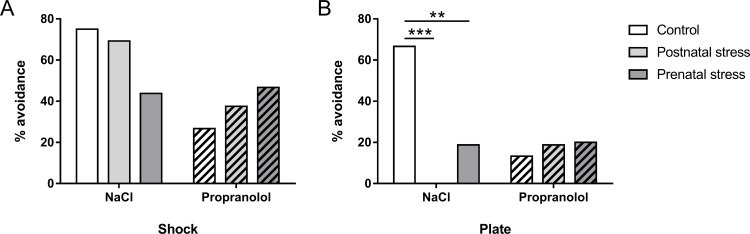
Effect of early stress on the avoidance behavior induced memory impairment in the “city-like” paradigm. Histograms correspond to the percentage of mice (+ SEM) that avoided the Shock (A) and the Plate (B) compartments during the test. **: p < .01; ***: p < .001 compared to NaCl group.

The first comparison was aimed at evidencing the effect of prenatal and postnatal stress on memory for the traumatic event in the “city-like” test in NaCl and Propranolol groups (Fig 9). As shown in Fig 9A, the percentage of mice that avoided the Shock compartment was similar between the three NaCl groups and between the three Propranolol groups suggesting that early stress did not affect the acquisition of FC for both conditions. Statistical analyses confirmed this observation and did not reveal any significant difference in the percentage of mice entering the Shock compartment for the different NaCl (F(2, 38) = 1.70; p = .197) and Propranolol groups (F(2, 43) = .62; p = .542).

As shown in Fig 9B, the percentage of mice that avoided the Plate compartment differed according to the type of stress condition. In the NaCl condition, the number of animals that avoided the Plate compartment was higher in the no-stress than in the prenatal and postnatal conditions. In contrast, propranolol injection did not affect the number of mice that avoided the Plate compartment. Statistical analyses confirmed this observation and revealed a significant effect of Stress in the number of mice that avoided the Plate compartment in NaCl condition (F(2, 38) = 10.96; p < .001) but not in the Propranolol condition (F(2, 43) = 0.12; p = .884). For NaCl group of mice, post hoc analysis demonstrated that Plate compartment is less avoided in postnatal (p < .001) and in prenatal (p < .01) mice compared to unstressed mice.

### Effect of the stress conditions on the freezing level in the FC test

**Fig 10:**
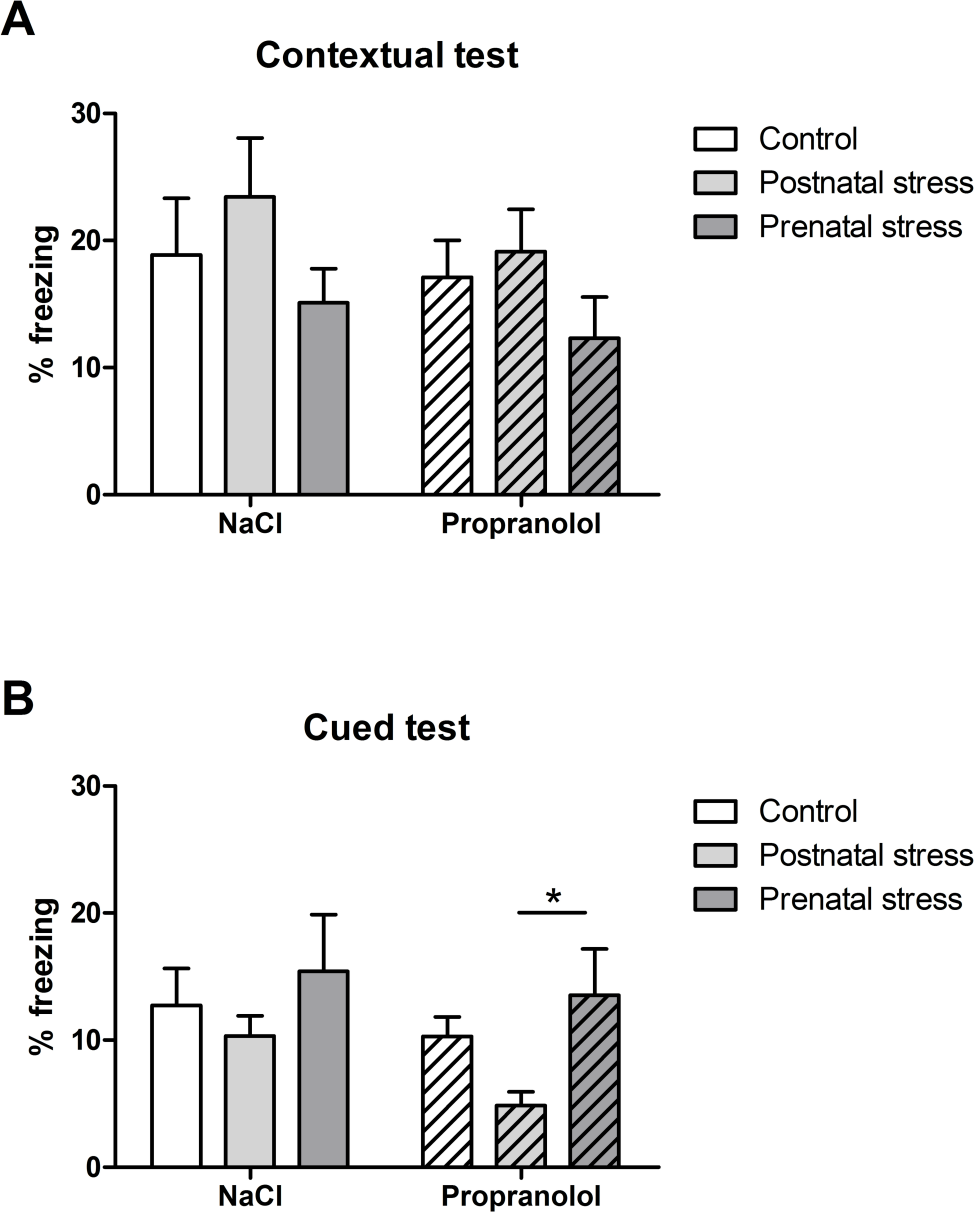
Effect of early stress on memory reconsolidation in the FC tests. Histograms correspond to total freezing time (mean + SEM) measured during the contextual test (A) and the cued test (B). *: p < .05 between Prenatal and Postnatal groups in the Propranolol condition.

In order to characterize the influence of stress on the effect of propranolol during the reconsolidation in the FC test, the subsequent analysis was aimed at comparing the percentage of freezing obtained in the different experimental groups during the contextual (Fig 10A) and cued (Fig 10B) versions of the FC test.

As shown in Fig 10A, the percentage of freezing obtained was similar between groups. Statistical analyses confirmed this observation and revealed no significant effect of factor Stress on the percentage of freezing in NaCl and Propranolol groups (F(2, 31) = 1.07; p = 0.35 and F(2, 34) = 1.23; p = .304 respectively) thus suggesting that early conditions did not influence the performance of contextual FC in the NaCl and Propranolol groups

In contrast, Fig 10B indicates that the percentage of freezing varied according to the type of Stress condition in the Propranolol but not in the NaCl group. More precisely, whereas animals in the NaCl groups showed a great amount of freezing during the presentation of the fear conditioned tone, animals in the Propranolol group that were subjected to postnatal stress showed a smaller amount of freezing during the cued FC test compared to their control. Statistical analyses confirmed these observations and showed a significant effect of factor Stress on the percentage of freezing in the Propranolol group (F(2, 34) = 3.27; p = 0.050) but not in the NaCl group (F(2, 31) = 0.64; p = 0.535). Interestingly, post hoc tests on Propranolol groups showed a significant decrease in the amount of freezing between pre- and postnatally stressed animals (p < .05) but not between postnatally and no-stressed groups.

Our results show that, in NaCl group of mice, prenatal and postnatal stress influenced the percentage of mice that avoided the Plate compartment but it is not the case for the Shock compartment. In the FC, early stress did not influence the level of freezing observed in the contextual and cued test. Moreover, our data show that in propranolol injected mice, early stress did not influence the percentage avoidance of the two compartments in the “city like” and the level of freezing for the contextual FC. However, even if the two groups of stressed mice are not different from the no-stressed group, we found a significant difference between the two groups of stressed mice on the freezing level in the cued FC. These results are summarized in Table 2.

**Table 2:** Summary of the stress effects (ANOVA) in the two behavioral tasks and in the two treatment groups.

*STRESS EFFECT*		NaCl	Propranolol
**City-like**	**Shock**	(F(2, 38) = 1.70; p = .197)	(F(2, 43) = .62; p = .542).
	**Plate**	**F(2, 38) = 10.96; p < .001**	(F(2, 43) = 0.12; p = .884
**Fear conditioning**	**Contextual test**	(F(2, 31) = 1.07; p = 0.35	F(2, 34) = 1.23; p = .304
	**Cued test**	F(2, 31) = 0.64; p = 0.535	**F(2, 34) = 3.27; p = 0.050**

Significant *P*-values are given in bold.

### Plasma corticosterone concentrations

#### Early stress influence on basal plasma corticosterone level

**Fig 11:**
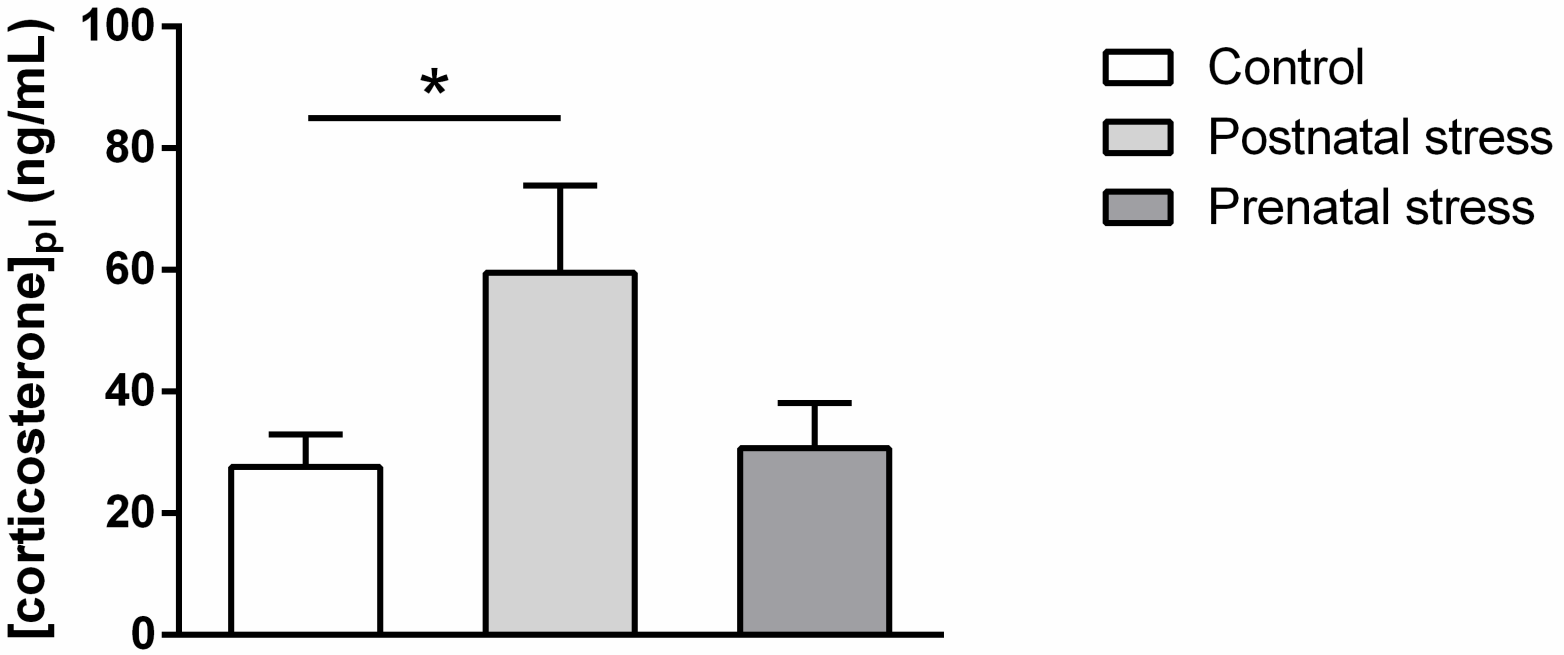
Plasma corticosterone concentration measured in 4 week-old mice in the different experimental stress conditions (mean + SEM). *: p < .05 compared to Postnatal stress group. (Control n = 12; Postnatal stress n = 9, Prenatal stress n = 10).

Fig 11 represents the plasma corticosterone concentrations measured 4 weeks after birth in each experimental group. It is important to note that plasma corticosterone level in the postnatal stress group was measured only two days after stress procedure. As shown in this figure, the plasma corticosterone concentration varied according to the treatment and only the group subjected to postnatal stress showed a higher corticosterone level. Statistical analysis confirmed this observation and revealed a significant effect of Stress (F(2, 28) = 3.56; p = .042). Post hoc analysis showed a significant difference in plasma corticosterone concentration only in postnatally stressed mice as compared to the Control (p = .048).

#### Effect of early stress on plasma corticosterone level in mice tested in the “city-like” paradigm

**Fig 12:**
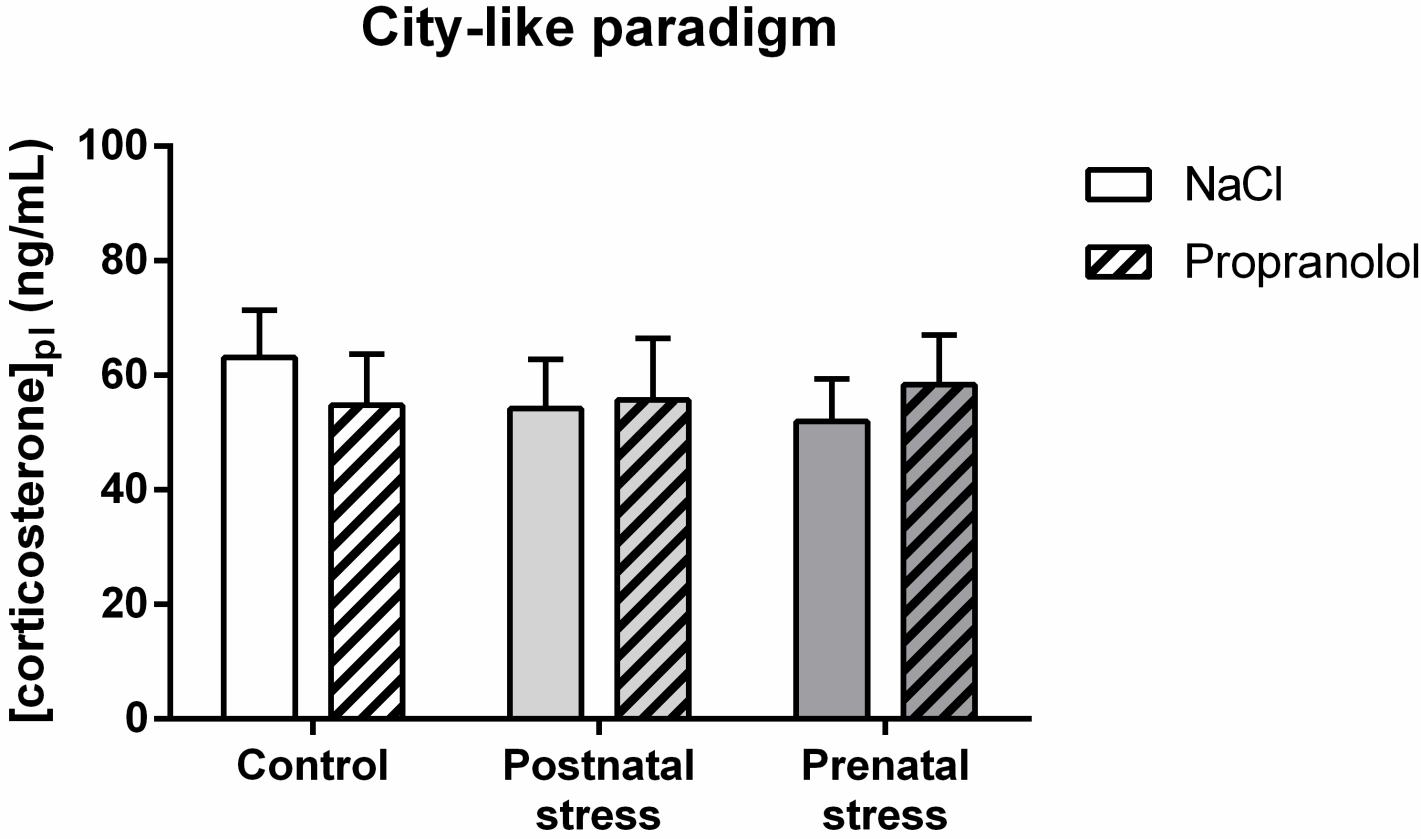
Effect of early stress influence on plasma corticosterone after the “city-like” test. Histograms represent plasma corticosterone concentrations (mean + SEM) in 8 week-old mice in the various groups.

Fig 12 represents the plasma corticosterone concentrations measured 30 min after the end of the “city-like” test in the various groups of animals. On this figure, the plasma corticosterone levels were similar and no difference was found between groups. Statistical analysis confirmed this observation and showed no significant effect of factor Stress (F(2, 85) = .13; p = .877), no significant effect of factor Treatment (F(1, 85) < 0.01; p = .987) and no interaction between those factors (F(2, 85) = .37; p = .693). These data suggest that early stress or propranolol injected during reconsolidation did not affect the level of stress measured in the animals 30 min after the end of the “city-like” test. However, the comparison between the level of corticosterone in the Control NaCl group (63.1 ng/mL) and the Control basal group (27.6 ng/mL, Fig 11) showed that testing the animals for traumatic memory greatly enhanced the level of stress.

#### Early stress effect on plasma corticosterone level in mice tested in the FC paradigm

**Fig 13:**
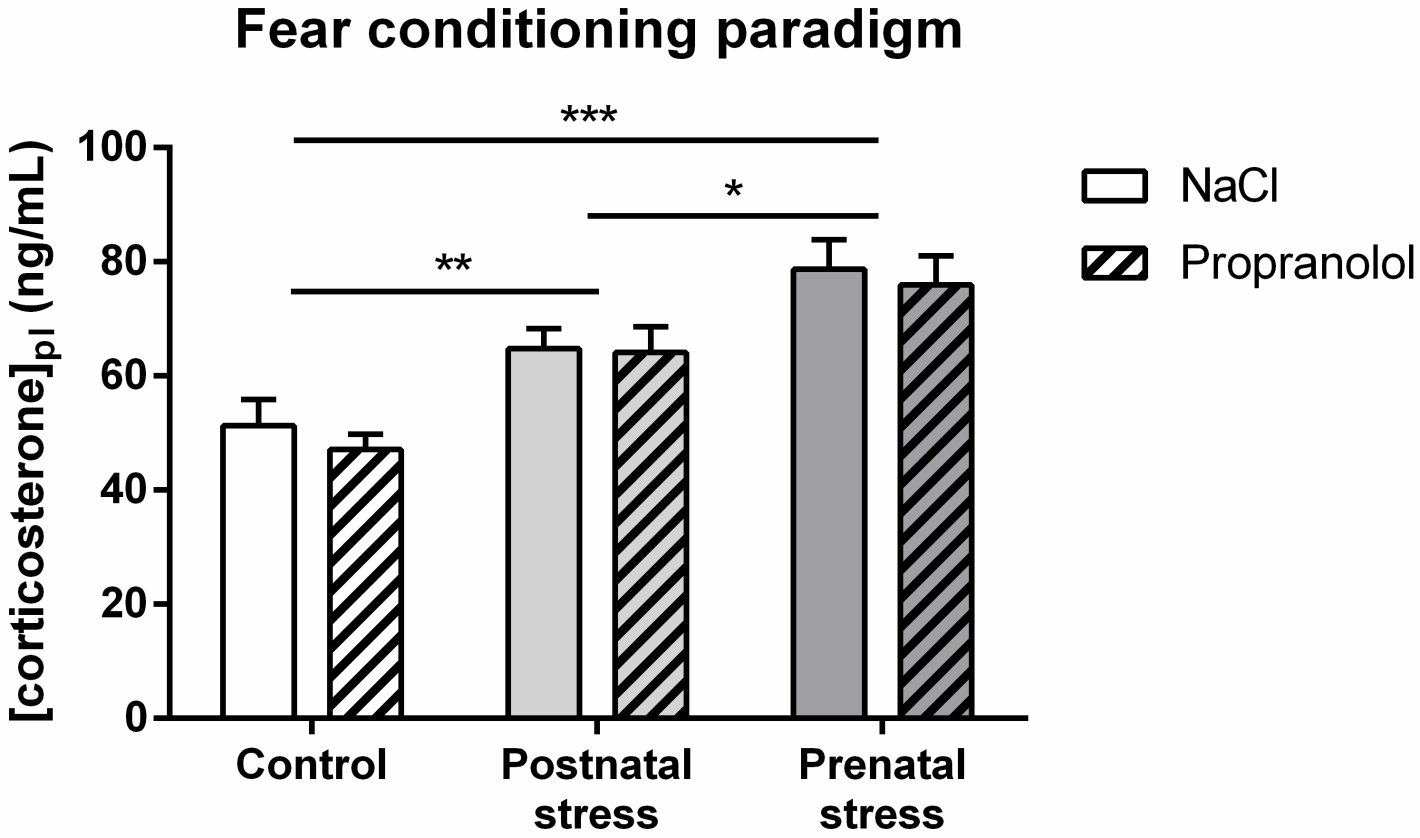
Effect of early stress influence on plasma corticosterone after FC tests. Histograms represent plasma corticosterone concentrations (mean + SEM) in 8 week-old mice in the various groups. *: p < .05 compared with Postnatal stress group, **: p < .01 and ***: p < .001 compared to the Control group.

Fig 13 represents the plasma corticosterone concentrations measured 30 min after the end of the FC test in the various groups of animals. On this figure, the plasma corticosterone levels varied according to the stress procedure but not according to the treatment. Whereas groups in the prenatal stress condition showed highest concentration of plasma, the group in the postnatal stress condition showed lower level of plasma concentration that was higher than this of the no-stress condition. Statistical analysis confirmed this observation and showed a significant effect of factor Stress (F(2, 68) = 20.59; p < .001), no significant effect of factor Treatment (F(1, 68) = .49; p = .487) and no interaction between those factors (F(2, 68) = .08; p = .923). Further post hoc analysis demonstrated a significant increase in plasma corticosterone concentration in postnatally stressed mice (p < .01) and prenatally stressed mice (p < .001) compared to the Control. In addition, post-hoc tests showed a significant difference in plasma corticosterone concentration between pre- and postnatally stressed mice (p < .05) but no difference between NaCl and Propranolol groups in the three stress conditions (p > .05). These data suggest that early stress affected the level of stress measured in the animals 30 min after the end of the FC tests.

In conclusion, postnatal stress increased plasma corticosterone concentration both at a basal level and at the adult age in the FC post-test condition, while prenatal stress increased this concentration only at the adult age after FC tests. In both tasks, propranolol injection did not change the plasma corticosterone concentration.

## Discussion

In the present “city-like” experiments, the different compartments were not similarly explored by mice during the test: control mice generally quickly entered the Neutral compartment not associated with shocks (within a few dozen seconds), but markedly avoided the Shock and Plate compartments. These observations suggest that the mice had strongly associated the shock trauma with the spatial location of the compartment and the plate cue where and through which the shocks were delivered. Times to enter the other compartments showed that control mice associated, although to a lesser extent, the olfactory, auditory and visual cues with the foot-shock, since they entered each compartment presenting each cue with a latency of 3 to 5 minutes).

Importantly, our “city-like” test was based on free exploration of the different compartments presenting the different cues previously associated with traumatic foot-shock, the mice choosing to expose themselves to these cues or not. Therefore, the various “percentage avoidance” and “latency to enter” variables represent the level of aversiveness previously acquired by each of the sensory cues during association with the US. By enabling investigation of the level of association of various types of sensory cue with the traumatic event, the ‘city-like’ paradigm is of particular relevance for the understanding of symptoms observed in PTSD patients, who avoid confrontation with places, objects or thoughts related to the trauma previously associated with them. In contrast to the classical used FC test where freezing is measured during direct forced confrontation with aversive contextual or cues with no possible escape, our “city-like” paradigm provided more refined measures related to the strength of aversion developed by the mice for each cues presented separately after the aversive conditioning.

In the no-stress condition, injection of propranolol during traumatic FC memory reactivation did not affect the reconsolidation process as already reported in the same paradigm [31]. However, in other studies, administration of propranolol at the time of retrieval was found to attenuate the strength of contextual and auditory fear memories in the literature [25,27].

Nonetheless, in our “city-like” test, propranolol injected after partial reactivation of conditioned fear memory in a new context presenting two of the four shock-associated cues, greatly reduced memory reconsolidation. Close observation of the effect revealed that propranolol clearly reduced percentage avoidance of the two compartments associated with the traumatic event (Shock and Plate) but only marginally affected latency to enter either compartment. While the selectivity of these effects has already been demonstrated in rats [24], the present results are not consistent with passive avoidance test data where propranolol affected both variables [20]. In the present results, partial reactivation in the “city-like” paradigm suggests that propranolol injected immediately after reactivation disrupted the memory process underlying reconsolidation, thus reducing the avoidance of shock associated compartments. Taken together, these results support the idea that our “city-like” paradigm provides a more subtle and behaviorally relevant experimental test for the study of memory processes leading to PTSD.

When mice were pre- or postnatally stressed, propranolol administered after partial reactivation in the “city-like” test affected neither latency nor percentage avoidance. However, avoidance levels in NaCl animals in the prenatal, postnatal and no-stress groups differed according to traumatic history. This was particularly evident for the Plate compartment, where the level of avoidance fell in the two groups of pre- and postnatally stressed mice. Why post- and especially prenatal stress decreases fear behavior in the “city-like” test in control mice is an important point. Unfortunately, we cannot answer this question clearly, but these kinds of result are sometimes found in different memory tasks and, in the 2012 review by Kosten et al. 2012 [82], different hypotheses were suggested to explain these results. Firstly, learning and memory performance may be altered by early life manipulations due, in part, to primary effects on unconditioned behaviors reflecting fear or anxiety. The majority of studies that assessed anxiety in the Elevated Plus Maze after a brief perinatal stress found that early life manipulation decreased anxiety [83,84]. Secondly, the duration of early life manipulation (stress induced by different durations of maternal separation) seems to be an important factor too: brief separation has been shown to enhance and prolonged separation to impair performance on spatial/relational tasks [85,86]. In addition, performance was impaired in aversive conditioning [70] but enhanced in inhibitory learning tasks regardless of manipulation duration [87]. Finally, the kind of behavioral measures (latency before moving, freezing, etc.) may explain differences in memory performances (increase or decrease; see [82] for a discussion of the importance of this parameter).

On the other hand, propranolol injected after reactivation failed to reduce avoidance in early-stressed animals, and avoidance of the Shock and Plate compartments was relatively similar between the prenatal, postnatal and no-stress groups. This lack of propranolol effect on avoidance level in the pre- and postnatally stressed groups may thus have been due to decreased avoidance in no-stress control animals more than to a lack of efficacy of propranolol in stressed animals. Actually, early stress resulted in a decrease of avoidance of the compartment where electric foot-shocks were administered. Thus, it is difficult to observe a decrease of memory performance after propranolol treatment in this condition. This is particularly true for the plate compartment. These decreases in level of avoidance are consistent with those obtained in the passive avoidance task, in which avoidance of the dark compartment (where animals received electric foot-shock through a plate or grid) was greatly reduced in postnatally stressed mice compared to non-stressed controls [70,88]. However, we could have expected a greater decrease in avoidance by combining the action of stress with propranolol injection. In control animals, there was still 70% and 40% avoidance of the shock compartment in the post- and prenatal stress groups respectively, and thus the level of avoidance could have decreased after treatment with propranolol. This was not the case; indeed, avoidance increased, although not significantly, in the 2 stress groups after propranolol treatment, which suggests that propranolol did not work for all groups equally, although of course, this hypothesis cannot be excluded.

In these experiments, we studied the effects of prenatal and postnatal stress separately; however, it might be interesting to compare the effect of a procedure combining pre- and postnatal stress to effects obtained with separate pre- and postnatal stress procedures in order to mimic in the complex PTSD symptoms observed in humans with repeated severe trauma during development.

Interestingly, the level of avoidance in NaCl mice was different between the no-stress and the two early-stress groups in the “city-like” paradigm, whereas the level of corticosterone was comparable whatever the traumatic history. Therefore, the decrease in avoidance level for the aversive Shock and Plate compartments does not seem to be due to a difference in the level of stress at the time of the behavioral test.

As for the FC test, comparison of freezing levels of the NaCl animals between the 3 groups showed that neither pre- nor postnatal stress influenced the level of freezing during the contextual and the cued versions of the test. These data are consistent with those supporting the meta-analysis published by Kosten et al. [82]. In this review, 6 of the 18 studies based on contextual fear conditioning paradigm reported no effect of stress, 10 reported impairment and 2 enhancement due to perinatal stress. In the same review, 8 of the articles describing the effect of perinatal stress on the cued fear-conditioning paradigm reported no effect, 4 reported impairments and 3 enhancement induced by stress. Interestingly, the absence of stress in the cued version of fear conditioning was mostly obtained in females, as was the case in our study.

Moreover, corticosterone levels were higher during the FC than during the “city-like” tests. These data show that the type of test directly influenced the level of corticosterone in early-stressed animals: free exploration of the different compartments in the “city-like” box, where mice could stay in a non-aversive place (Neutral compartment), was a less stressful experience than the situation where they could not escape the presentation of conditioned aversive context and cues in the FC. These results are consistent with the literature, in which postnatal stress is reported to increase corticosterone levels in both baseline and stressful conditions in rats, with faster return to normal after stress (see [89] for review). Comparison between results in the two versions of the FC test showed that, whereas propranolol did not affect the level of freezing in any of the 3 pre-, postnatal and no-stress groups in the contextual test, β-adrenoceptor blockade induced a decrease in percentage of freezing in the post- but not in prenatally stressed group during the cued fear conditioning test. This lack of propranolol effect in prenatally stressed animals was rather unexpected in the light of the literature but it may be that, when the initial level of stress is too high, as evidenced by the very high corticosterone level observed in this group during FC, propranolol no longer has an effect on avoidance.

Related to this, some studies have reported that acute stress induced an increase in circulating corticosterone concentration that directly affected learning and memory retrieval performance in rats [61,90]. Moreover, this effect was shown to depend on β-noradrenergic activation of the amygdala (review in [91]). Numerous other studies also highlighted a strong relationship between stress, corticosterone level and the noradrenergic system. For example, Aisa et al., in 2007 [92], showed that long after postnatal stress (e.g. maternal separation), rats presented a memory deficit in a new object-recognition task and a higher level of plasma corticosterone in response to an acute stressor such as maternal separation. Moreover, the negative effect of the early stress on object recognition memory was reversed by injection of propranolol. In other studies in rats, propranolol was shown to block the impairing effects both of corticosterone on working memory [93] and of intra-hippocampal administration of the glucocorticoid receptor agonist RU 28362 on memory retrieval [61]. However, in all these studies, propranolol was injected just before the behavioral task; thus the animals were under noradrenergic antagonist influence during the test. This was not the case in our study, where propranolol was injected during memory reactivation, as in PTSD treatment in humans. Consequently, animals were never tested under the acute influence of propranolol but rather the day after injection, when the drug was no longer present in the organism.

## Conclusion

Our new “city-like” behavioral paradigm enables measurement of one of the major symptoms observed in PTSD patients: avoidance of places recalling the traumatic event. Moreover, and in contrast to what was observed in the fear conditioning task, propranolol injection immediately after reactivation strongly affected reconsolidation of the traumatic memory. On the other hand, even though propranolol did not affect memory reconsolidation in pre- or postnatally stressed mice, it is difficult to demonstrate unambiguously that early stress is a factor of resistance to propranolol treatment, because of the high level of avoidance found in early-stressed NaCl mice. Moreover, the present study showed that propranolol did not decrease post-test corticosterone levels, whereas it strongly decreased the avoidance variables. These results seem to indicate that propranolol does not act on general stress but only on the traumatic memory reconsolidation. By revealing a significant effect of propranolol on one of the major symptoms of PTSD, (i.e., avoidance of particular cues), our results demonstrated that the “city-like” paradigm is more suitable and relevant than FC for investigating the role of the -β-adrenergic system in the processes underlying traumatic memory reconsolidation and opens the way for new therapeutic perspectives in research on PTSD treatment.

## Supporting information

S1 Dataset Manuscript dataset. The first sheet of the document provides the percentage of freezing during contextual and cued fear conditioning tests. Sheets 2 and 3 provide latencies (in sec) before entering each compartments during respectively the familiarization and the test phases of the “city-like” experiment, and sheet 4 provides the total duration (in sec) spent in each compartment during the “city-like” test. Sheet 5 shows the different concentrations of plasmatic corticosterone (in ng/mL).
(XLSX)
